# Use of ‘Mishri’ A Smokeless form of Tobacco During Pregnancy and its Perinatal Outcome

**DOI:** 10.4103/0970-0218.62547

**Published:** 2010-01

**Authors:** Asha Pratinidhi, Sudesh Gandham, Aparna Shrotri, Archana Patil, Shrikar Pardeshi

**Affiliations:** Director of Research, KIMS University, Karad, India; 1 Associate Professor, Department of PSM, RCSM GMC, Kolhapur, India; 2 Ex-Professor, Department of Obstetrics/Gynecology BJMC, Pune, India; 3 Deputy Director (PDE) DHS, Mumbai, India; 4 Ex.Lecturer, Department of PSM, BJMC Pune, India

**Keywords:** Smokeless Tobacco, perinatal outcome, Mishri – tobacco containing teeth cleaning powder, stopping consumption of tobacco

## Abstract

**Background:**

Use of ‘Mishri’ (Tobacco containing teeth cleaning powder) is common in the central and southern part of India.

**Objectives:**

To study the effects of Mishri use on the fetus during pregnancy and the perinatal outcome, and stopping its use.

**Materials and Methods:**

All apparently healthy pregnant women were enrolled at 20 weeks of gestation from rural Maharashtra, India. Information related to use and giving up of Mishri, previous obstetrical history, current pregnancy, delivery and outcome during the perinatal period were recorded. Appropriate tests of significance were applied.

**Results:**

Out of 705 enrolled pregnant women, 218 (30.9%) were using Mishri. The proportion of women with complications during the previous perinatal period, complaints and complications during the current pregnancy/delivery and the number of stillbirths were significantly more among Mishri users. A relative risk of abnormal delivery was 2.7 for the users. In spite of counseling, 153 women never stopped the use of Mishri and gave birth to babies weighing on an average 169.9 gm less (statistically significant) than babies born from the group that never used it. Babies of 28.8% who stopped/reduced consumption of Mishri were significantly benefited.

**Conclusions:**

The improvement seen in babies born to 28.8% mothers who stopped/reduced consumption of Mishri by 32 weeks during the current pregnancy is of paramount importance in the developing world for primary prevention of low birth weight.

## Introduction

Although smoking by women is not well accepted in Indian society, consumption of smokeless tobacco is well accepted, and use of Mishri (tobacco containing teeth cleaning powder) is very common. Various studies have estimated the prevalence of the use of Mishri from 17 to 45%(1–10) It is a known fact that smoking during pregnancy leads to higher incidence of Low Birth Weight (LBW).(11–13) Incidence of antepartum hemorrhage, placental abruption, placenta previa, and premature rupture of membranes is also high in women who smoke during pregnancy. Mishri is prepared by roasting tobacco leaves, principal constituent being alkaloid nicotine in 1 to 7%. The roasted tobacco leaves are powered and it is known by various names like ‘Mishri,’ ‘Masherior,’ ‘Misheri’. It is mainly a homemade preparation, but is also available in the market under different names. It does not contain anything other than tobacco leaves.

Considering a high prevalence of Mishri use by women and the possible harmful effects on the growing fetus, a study was planned on the use of smokeless tobacco by pregnant mothers and its effect on the baby.

This project received financial support from the Ministry of Health and Family Welfare, Government of India.

## Materials and Methods

A community-based interventional study was started in the Primary Health Centers, ‘Kunjirwadi’ and ‘Yawat’ of Pune District, in the rural areas of Maharashtra, India.

All apparently healthy pregnant women of 20 weeks gestation were enrolled. Any woman with any known major illness, multiple pregnancies, as well as those who had planned their delivery outside the study area were excluded from the study. Thus a total of 705 eligible pregnant women were enrolled for the study from Mach 2003 till July 2005, for a period of 29 months and followed up till December 2005 (i.e., till the last enrolled woman delivered). Modular training of two days was given to all health workers explaining the ill effects of tobacco.

A group meeting was organized in every village and hamlet and women folk were explained the purpose of study and the methodology. A written consent was taken from all the eligible pregnant women in the vernacular language before collecting information. If a woman did not attend the antenatal clinic, antenatal care was given at home and consent was taken at home after explanation similar to the one given in the group meeting.

In addition to routine antenatal care (ANC), all pregnant women were motivated to give up the use of Mishri for the benefit of the growing fetus.

Counseling about the ill effects of tobacco on the health of the woman and her growing fetus was undertaken every time the woman was examined antenatally either at the clinic or at home. Every pregnant woman using ‘Mishri’ had a minimum of three such sessions.

All information related to the use of Mishri, previous obstetrical history, current pregnancy, and the delivery and its outcome, during the perinatal period, were recorded on a pretested proforma. Information about the duration and frequency of the use of Mishri was collected. It was not possible to measure the amount of ‘Mishri’ consumed by each mother, but it was possible to recognize whether she had stopped using ‘Mishri’ completely or reduced the frequency of its use. Those who never used Mishri were graded as having no exposure, those who stopped at 28 weeks as having low exposure, those who stopped at 32 weeks or reduced frequency of use as moderate exposure, and those who did not change their habit as high exposure, as they were a hard core group and so addicted that they could not reduce or give up the use of tobacco. Analysis was done by applying appropriate tests of significance.

## Results

In all 705 pregnant women were enrolled from March 2003 to July 2005 and followed up from 20 weeks of gestation till delivery. By December 2005 all enrolled women had delivered. There were 343 women enrolled from Primary Health Centere (PHC) Kunjeerwadi and 362 from PHC Yawat. Out of them 218 (30.9%) were using tobacco containing teeth cleaning powder (Mishri) and 487 (69.1%) did not use tobacco in any form. The profiles of pregnant women using Mishri and not using Mishri are given in Table 1.

**Table 1:** Comparison of profiles of Mishri users and non users

Characteristics	Mishri users (n = 218)		Non users (n = 487)		Z	*P* value
				
	No.	%	No.	%
Maternal age < 20 years	61	28	179	36.8	2.4	< 0.05*
Parity I	57	26.1	175	35.9	2.6	< 0.05*
Inter-pregnancy interval < 24 months	36	22.3	72	23.1	0.2	> 0.05
Occupation of wife as housewife	194	88.9	438	89.9	0.4	> 0.05
Illiteracy	67	30.7	44	9.0	6.5	< 0.05*
Per capita income < Rs. 500 per month	142	65.1	316	64.9	0.05	> 0.05
Caloric intake < 1500 calories/day	86	39.5	180	36.9	0.6	> 0.05
Protein intake < 40g/day	201	92.2	441	90.6	0.6	> 0.05
Maternal height < 145 cm	43	19.7	93	19.1	0.2	> 0.05
Maternal weight at 28 weeks < 45 kg	67	30.7	186	38.2	2.0	< 0.05*
Maternal Hb < 8 g	22	11.1	42	8.6	0.6	> 0.05

* Statistically significant

Biologically predisposing factors such as maternal age less than 20 years, primi parity, and maternal weight less than 45 kg at 28 weeks, were significantly higher among the nonuser group. In spite of this, significantly higher low birth weight babies were seen in users as compared to nonusers.

Factors such as occupation of wife, per capita income, caloric and protein intake during pregnancy, inter-pregnancy interval, maternal height, maternal hemoglobin level, and sex of the newborn were comparable in the two groups.

Only one sociocultural risk factor that was significantly higher among the user group was illiteracy.

It was seen that the rate of complications during the previous pregnancies were higher among the current Mishri users than in non-users [Table 2]. History of spontaneous abortions was the most frequent complication as reported by 9.6% users and 6.3% nonusers. Users had a significantly higher relative risk of LBW and prematurity.

**Table 2:** Previous perinatal history among Mishri users and non users

Obstetrical history	Users (n = 218)		Non users (n = 487)		RR*	95% CI** for R.R. 95% CI
				
	No.	%	No.	%
H/O Spontaneous abortion	21	9.6	1.5	31	6.3	0.84–2.67
H/O LBW	24	11.0	2.1	25	5.1	1.17–3.77***
H/O Prematurity	14	6.4	2.2	14	2.8	1.03–4.69***
H/O Early neonatal deaths	4	1.8	2.2	4	0.8	0.55–8.88
H/O PPH	1	0.4	2.2	1	0.2	0.14–16.06

* RR Relative risk,

** Confidence Interval,

*** Statistically significant

It was evident that women using Mishri had significantly higher untoward outcomes in the current pregnancy than the non-Mishri users. The proportion of women having complaints during pregnancy, complications during delivery, proportion of low birth weight babies, and stillbirths [Table 3] were significantly higher among the Mishri users as compared to the non-users.

**Table 3:** Current pregnancy outcome and Mishri use

Characteristics	Mishsri users (n = 218)		Non users (n = 487)		χ^2^	*P* value
				
	No.	%	No.	%
Complaints during pregnancy	65	29.8	111	22.8	3.9	<0.05*
Complications during pregnancy	46	21.1	42	8.6	12.4	<0.05*
Operative deliveries	23	10.6	19	3.9	11.8	<0.05*
Low birth weight	42	19.3	44	9.0	14.6	<0.05*
Preterm births	21	9.6	35	7.1	1.2	>0.05
Stillbirths	6	2.7	3	0.6	5.4	<0.05*
Early neonatal deaths	8	4.7	8	1.7	2.9	>0.05

* Statistically significant

Out of a total 705 women enrolled in this study, 176 (25%) women had one or the other complaint during the present pregnancy, common ones being anorexia, nausea, vomiting, weakness, swelling over legs, low backache, acidity, white discharge, giddiness, and so on. The proportion of women having complaints was higher in the users 65 (29.8%) than in the non-users 111 (22.8%), and this difference was statistically significant. (χ^2^ = 3.9; *P*<0.05)

The rate of all the complications, except oligohydramnios, was higher in users (21.1%) than in non-users (8.6%) [Table 4]. Fetal distress was the most common and significant complication that was seen in 31 (14.2%) users and 30 (6.1%) nonusers. The relative risk of 5.5 was significant and the highest for pregnancy-induced hypertension.

**Table 4:** Complications during delivery and Mishri use

Complications during delivery	Users		Non users		Relative risk	95% CI** for R.R.
				
	No.	%	No.	%
Fetal distress	31	14.2	30	6.1	1.8	1.06 - 3.06***
Pregnancy induced hypertension (PIH)	5	2.3	2	0.4	5.5	1.06 - 28.57***
Premature rupture of membranes (PROM)	4	1.8	0	0.0	-	-
Antepartum hemorrhage (APH)	3	1.3	5	1.0	1.3	0.30 - 5.49
Polyhydramnios	1	0.4	1	0.2	2.2	0.14-35.33
Oligohydramnios	0	0.0	1	0.2	-	
Post partum hemorrhage (PPH)	2	0.9	3	0.6	1.4	0.23 - 8.44
Total	46	21.1	42	8.6	2.1	1.33 - 3.31

*** Statistically significant

A higher proportion of nonusers (96.1%) had a normal vaginal delivery as compared to users (89.4%). There were 23 (10.6%) operative deliveries among the Mishri users as compared to 19 (3.9%) among the nonusers. This difference was statistically significant (χ^2^ = 10.1, *P*<0.05), the relative risk of operative delivery (forceps, ventouse, LSCS) for Mishri users was 2.7, with a confidence interval of 1.46 to 27.94.

Duration of use of < 2 years (22%) was associated with significantly higher (Z = 2.63) mean birth weights (2698.5 g) than those who used it for six years or more (31.7%), with mean birth weights of (2570.1g). Those who used it for more than two years, but less than 6 years (46.3%) did not differ significantly from its use of a shorter or longer duration.

14.7% used Mishri only once, 44.5% twice, 22.9% thrice, and 17.9%, four times or more. Frequency of use was not associated with significant differences in the mean birth weights.

Intensive health education was undertaken on a one-to-one basis, as well as group counseling. Various charts were used to explain the ill effects of tobacco, with special reference to the growing fetus.

Out of 218 (30.9%) women using Mishri, only 29 (13.3%) stopped use of smokeless tobacco by 28 weeks of gestation. Thirty-six (16.5%) reduced consumption or stopped at 32 weeks, and the remaining 153 (70.2%) did not change their habit of using Mishri at all.

It was seen that the mean birth weight was highest for babies born to the ‘never used’ group, followed by those who ‘stopped Mishri use by 28 weeks,’ followed by those who ‘reduced consumption or stopped its use late in pregnancy by 32 weeks,’ and it was lowest in the group whose ‘Mishri’ use was unabated [Table 5]. There was no significant difference between the mean birth weights of babies born to the first three groups, but all of them differed significantly with the ‘never stopped’ group. The 153 women who never stopped the use of Mishri gave birth to babies weighing, on an average, 169.9g less (statistically significant) than the ‘never used group’.

**Table 5:** Effect of counseling on Mishri consumption and mean birth weight

Group and exposure status	Number	%	MBW*	SD
Never used (No exposure)	487	69.1	2750.3	344.0
Used and completely stopped at 28 weeks (Low exposure group)	29	4.1	2736.2	272.3
Used and stopped at 32 weeks or reduced consumption (Moderate exposure group)	36	5.1	2708.8	210.6
Never stopped (High exposure group)	153	21.7	2580.4	275.6
Total	705	100.0	2710.0	313.9

* Z values for mean birth weights were as follows: 1) Never used never stopped (significant) Z = 6.2, *P* < 0.05. 2) Stopped at 28 weeks vs. never stopped (significant) Z = 2.81, *P* < 0.05. 3) Reduced consumption or stopped late by 32 weeks vs. never stopped. (Significant) = Z 3.08, *P* < 0.05

## Discussion

The proportion of women using Mishri in this study of 30.9% is comparable to others studies.(1–10)

The profile of Mishri users and Non-Mishri users was similar, except for the level of literacy, proportion of teenage pregnancies, proportion of primiparity, and the average maternal weight at 28 weeks [Table 1]. Difference in educational status was not surprising as the use of Mishri was more common among the illiterate population. The lower proportion of women, with age less than 20 years and primiparity, among Mishri users, could be due to a higher proportion of women experiencing obstetrical mishaps during their previous pregnancies. A higher proportion of women with lower body mass among non-users, could be related to the higher proportion of teenage mothers.

In the present study bad obstetrical history and complications during the current pregnancy were associated with current Mishri use [Tables 2–4].

Reliable information about the use of Mishri at the time of occurrence of an untoward event in the past was not possible, but considering the short inter-pregnancy interval and prolonged use of Mishri by women folk, it was very likely that the untoward outcome in the previous pregnancy was also related to the use of Mishri. However, this aspect of repeated untoward outcomes could be tested in a community-based prospective study of a longer duration. The nicotine caused decreased blood supply, reducing both oxygen and nutrition supplies to the uterus and placenta, hence the incidence of spontaneous abortion, placental abruptions, premature rupture of membranes, and fetal distress is high in women who smoke.(14–17) Nicotine also retards intrauterine growth, and could cause preterm deliveries and stillbirths.(17)

The high proportion of operative deliveries among Mishri users could probably be due to a higher rate of complications during pregnancy and delivery.

There were 4.7% early neonatal deaths in the users as compared to 1.7% in the nonusers [Table 3]. This difference in the proportion of early neonatal deaths and survivors in the two groups was not significant statistically, although smoking could increase the risk of neonatal deaths. This was observed among babies of smokers(20) The mean birth weight of the babies born to unabated Mishri users was 2580.4gm. which was 169.9 g lower (statistically significant) than the mean birth weight of babies born to the ‘never used’ group.

There are many studies on the use of tobacco during pregnancy (smokable forms) and its adverse effects on the birth weight of the baby.(17–20) Indian workers have found 100 – 450 g lesser values of mean birth weights of the babies born to Mishri users as compared to non-users.(6–8,10) Our results are in this range. There is a wide spread belief that topical use of tobacco improves oral hygiene, prevents dental carries, and because the tobacco is not swallowed there are no adverse systemic side effects. As, there is a social sanction for the use of tobacco in the form of ‘Mishri’, the habit is very often inculcated by parents to the offsprings from early childhood itself. As this practice of the use of Mishri is deeply rooted in the families, homemade preparations are very common. Very often Mishri is applied more than once to the gums and teeth and retained in the mouth for a period of 10 to 15 minutes before it is washed, allowing absorption of a substantial amount of active ingredients, principally nicotine.

The finding in this study that babies born to those who reduced or stopped the use of Mishri had mean birth weights similar to those who never used Mishri, is of paramount importance.

The decreasing trend of mean birth weights with increased exposure to Mishri is very clear and indicates dose response. Although the actual amount of mishri used by the pregnant women could not be measured, grading of exposure was possible by finding out the time of stopping or reduction in consumption of Mishri [Table 5]. A similar observation was made among smokers. The babies born to smokers who abstained for the last half of the pregnancy were of the same birth weight as those babies born to non-smokers.

Primary prevention of the use of tobacco is definitely desirable and is a long-term objective, although difficult to achieve. Those who are habituated to the use of Mishri, at least some of them may, with intensive health education and counseling, give it up or reduce its use during pregnancy, with a favorable outcome of pregnancy. In this study, over 70% of the mothers using smokeless tobacco did not respond to the health educational drive. It is not surprising, as tobacco is a psychoactive and addiction causing substance and it is very difficult to stop using it, be it the smoking or the smokeless form.

Wide dissemination of knowledge and awareness generation programs for all adolescents and women folk in general and pregnant mothers in particular may go a long way in reducing this dreaded habit of Mishri use, thereby reducing the risk of low birth weight and adverse perinatal outcome in the babies born to them.
